# Six Years of Experience in Photodynamic Therapy for Basal Cell Carcinoma: Results and Fluorescence Diagnosis from 191 Lesions

**DOI:** 10.1155/2014/849248

**Published:** 2014-09-14

**Authors:** M. Fernández-Guarino, A. Harto, B. Pérez-García, A. Royuela, P. Jaén

**Affiliations:** ^1^Dermatology Department, Ramon y Cajal Universitary Hospital, Carretera de Colmenar Km 9,100, 28034 Madrid, Spain; ^2^Statistics Department, Ramon y Cajal Universitary Hospital, Carretera de Colmenar Km 9,100, 28034 Madrid, Spain

## Abstract

*Background.* Photodynamic therapy (PDT) has become a therapeutic option for basal cell carcinoma (BCC) in the last decade*. Objectives.* To study the results and predictors of BCC response to treatment with PDT and to evaluate fluorescence diagnosis of BCC.* Methods.* A descriptive, retrospective, and observational study was carried out. Patients with biopsy-confirmed BCC who were treated with methyl aminolevulinate and red light according to standard treatment protocols (2 sessions separated by 2 weeks, 630 nm, 37 J/cm^2^, 8 minutes, Aktilite) were selected. Response was scored as clinically complete and incomplete and the patients were followed up every three months.* Results*. Data from 191 BCC in 181 patients with a mean age of 69.55 years and a mean follow-up period of 34.4 months were collected. The overall response was 74% of the BCC treated, with the best response in superficial BCC with a 95% of complete response. The regression analysis revealed that the superficial histological type was the primary factor predictive of a complete response.* Conclusions.* In the treatment of BCC with PDT, the most significant factor for predicting response is the histological type.

## 1. Introduction

PDT with MAL was approved in Europe in 2005 for the treatment of superficial (sBCC) and nodular (nBCC) basal cell carcinoma (BCC) [1]. The results of PDT on BCC have been evaluated in several studies, most of them clinical trials. The cure rates achieved in these studies were 80–90% for sBCC [2–5] and 52–73% for nBCC [2, 3, 6, 7]. The level of recommendation in sBCC treatment guidelines is A with a level of evidence of I and B for nBCC with a level of evidence of I (surgery continues to be the gold standard for nBCC) [8]. However, since its approval, few large retrospective studies that study the results of its daily use and on fluorescence diagnosis have been published [9–11]. These studies, though having less statistical power than the clinical trials, reveal new aspects of PDT on BCC by describing what happens in routine clinical practice. This study summarizes the findings of six years of experience in the treatment and fluorescence diagnosis using PDT on BCC in a series of 181 patients and also provides a long follow-up period.

## 2. Materials and Methods

A descriptive, retrospective observational study was carried out between May of 2005 and May of 2011. Data from patients with BCC treated with PDT were collected from three dermatologists at the same center. All of the office visits from the three dermatologists' schedules were collected using an Excel spreadsheet and patients diagnosed with BCC by skin biopsy, cross-checked with the pathology database from the same hospital (“Cajal” program), were selected. Cases that lacked sufficient clinical and photographic follow-up were excluded. Patients who were treated for more than two BCCs were also excluded in order to avoid inclusion of extraneous variables or a hypothetical case of undiagnosed Gorlin syndrome.

Only patients who received conventional two-session treatment were selected [1]. Methyl aminolevulinic acid (MAL) was occluded for 3 hours followed by illumination with red 630 nm light at 37 J/cm^2^ for 7 minutes (Aktilite). The BCC lesions were only subjected to cleaning with gauze and saline solution prior to application of MAL and nBCC lesions also underwent light and superficial curettage as defined in treatment guidelines [1].

Data on the patient's age and gender, BCC histological type, response, location and size of the lesion, fluorescence, and follow-up period were collected. The histological type was determined by the pathologist and divided into superficial, nodular, infiltrating, sclerodermiform, and not specified. The locations were divided into head (face and neck) and body. Locations on the face were specified in different zones: scalp, forehead, temple, nose, cheeks, earlobes, upper lip, and chin-mandible. The nose was also subdivided into the base of the nose, nostril, and tip of the nose. The response was classified as complete or incomplete and in the case of the latter, it was specified whether subsequent simple surgery or Mohs surgery was required. The size of the lesions was measured using the Photoshop “ruler” tool, which allows for the maximum diameter to be calculated using clinical photographs, and they were classified into three categories: 1: ≤1 cm; 2: 1-2 cm; 3: ≥2 cm. The fluorescence photographs were taken using an Olympus C5060 camera with ultraviolet flashes (Clearstone). The fluorescence of the lesions was classified as negative or positive. When the lesion was positive, it was classified in relation to the clinical margin appreciated by the dermatologist. The positive fluorescence was divided into excessive, exact, or defective, based on whether it extended beyond the clinical edge of the lesion, it perfectly delineated the lesion, or it did not reach the clinical edge of the lesion.

Statistical analysis of the data was carried out using SPSS. The chi-squared test was used for contingency analysis of the variables for histological type, location, size, fluorescence, and response. The interaction between all of the variables was quantified using logistical regression analysis.

## 3. Results

**Figure 1:**
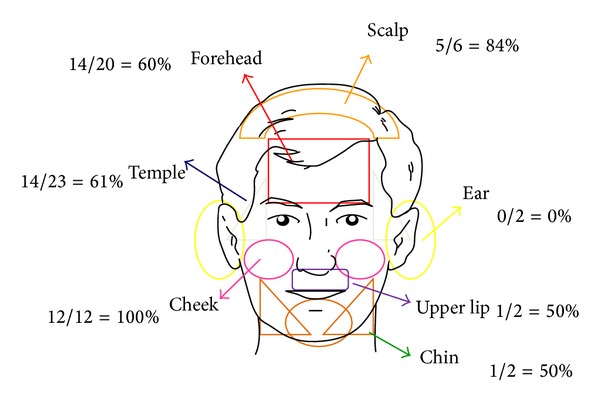
Number of BCC, location, and response in the head.

A total of 191 BCCs were collected (see Table 1), 110 in men and 81 in women, from 181 patients with a mean age of 69.55 years (range 34–98). Of the 191 BCCs, 73 (38%) were located on the body and 118 (62%) on the head with the following distribution: 44 on the nose (25 on the tip of the nose, 12 at the base of the nose, and 7 on the nostril), 20 on the forehead, 2 on the chin, 7 on the neck, 2 on the ears, 2 on the lip, 12 on the cheek, 23 on the temple, and 6 on the scalp (see Figure 1). Regarding the histological type, as shown in Table 1, 87 (46%) were superficial, 49 (26%) were nodular, 31 (16%) were not specified, 22 (11%) were infiltrating, and 2 (1%) were sclerosing. Regarding the distribution, the majority of the sBCCs (57%) were located on the trunk and the majority of nBCCs were located on the head (*P* < 0.001). The size of the BCCs collected was less than 1 cm in 60 (31%) and 1 to 2 cm in 99 (52%), and 32 (17%) were greater than 2 cm. Once again, the distribution is not random but rather statistically significant (*P* < 0.001). The BCCs smaller than 1 cm that were treated were more frequent on the head (92%), while the larger treated lesions were more frequent on the trunk. Fluorescence was exact in 98 (52%) of lesions, excessive in 80 (41%), and defective in 13 (7%). Negative fluorescence was not observed in any cases. Exact fluorescence is much more frequent on the trunk (*P* < 0.000, 65%) than the head, where excessive fluorescence was more frequent (95%).

The response was complete in 141 lesions and incomplete in 50, which translates to an overall response rate of 74% (141/191). The mean follow-up period for the BCCs in complete response (CR) was 34.4 months (range 6–72 months). Of the 50 BCCs that did not respond to PDT, 48 were surgically extirpated (28 of these 48 using Mohs surgery) and the other two were considered inoperable. Curiously, all of the incomplete responses (IR) or recurrences after treatment that required subsequent extirpation occurred in the first 6 months after PDT sessions.

The responses based on location, histological type, size, and fluorescence are described in Table 2. It is noted that the BCCs treated on the trunk responded better than those on the head, 86% versus 66%, respectively (*P* < 0.001). Superficial BCC responds best to PDT with a 95% complete response rate and this response is statistically significant (*P* < 0.001) versus the other histological types. Nodular BCC had a complete response in 49% of cases, not specified types in 71%, infiltrating in 50%, and neither of the two morpheaform BCCs treated responded to treatment. The same table (see Table 2) reveals that no association was found between the size of the BCC and the response to treatment (*P* < 0.063). BCCs that showed exact fluorescence achieved better response rates (*P* < 0.029).

Figure 1 shows the results obtained in the face. In this case, the small sample size did not allow for inferential statistical analysis to be carried out, but we can see the tendencies. There are areas that have a very good response, such as the check and the scalp with 100% and 84% of complete responses, respectively. Areas with an intermediate response such as the temple, forehead, upper lip, and chin had complete response rates of 61%, 60%, 50%, and 50%, respectively. The ear was an area of poor response to treatment with no complete responses, though only two BCCs were treated in this area. Figures 3, 4 and 5 shows the results of some patients treated.

**Figure 2:**
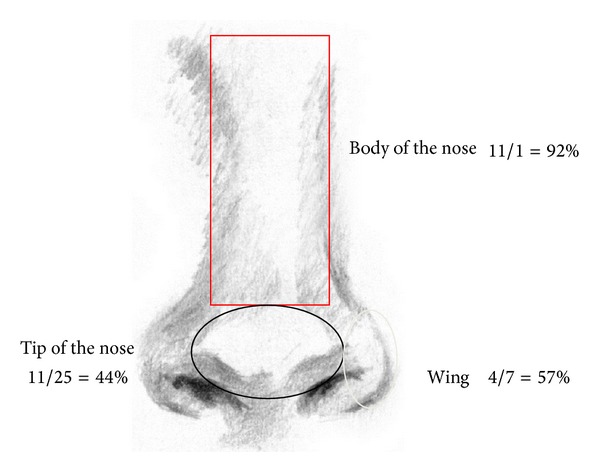
Location and response of the BCC in the nose.

**Figure 3:**
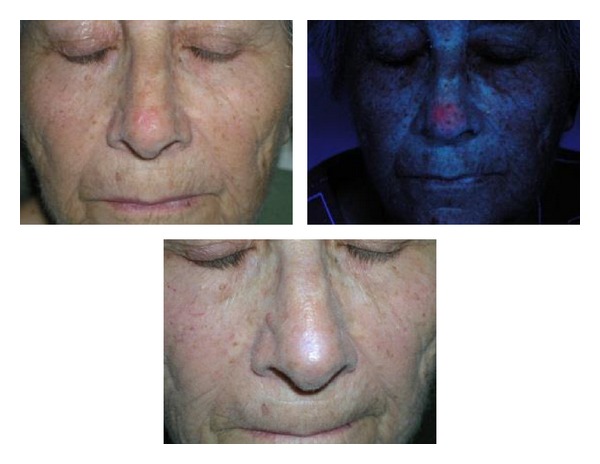
Superficial BCC in the nose. Inexact fluorescence and complete response (24 months).

**Figure 4:**
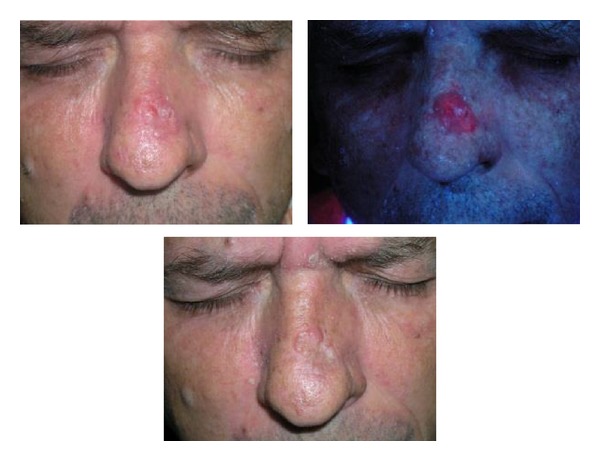
Nodular BCC in the nose. Exact fluorescence and incomplete response.

**Figure 5:**
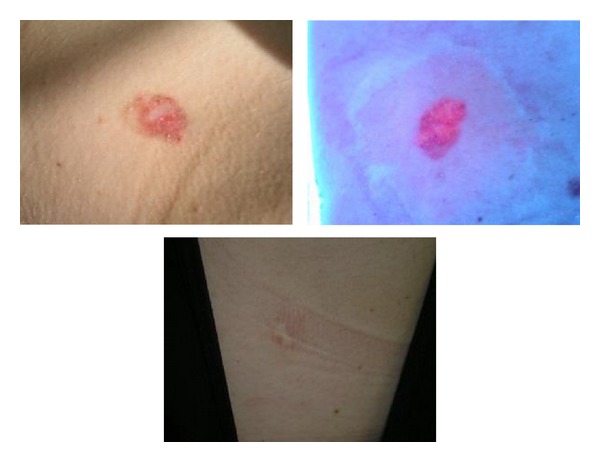
Superficial BCC in the lower limb, exact fluorescence, and complete response (72 months).

Figure 2 shows the results obtained on the nose. The response on the body of the nose is greater, 92%, than on the tip of the nose or the nostril with 44 and 57%, respectively.

Table 3 shows the fluorescence pattern of the lesions studied. Fluorescence was statistically more precise on the trunk (*P* < 0.000), more precise in sBCC (*P* < 0.000), and more precise in large BCCs, sizes 2 and 3 (*P* < 0.000).

A logistical regression analysis was carried out in order to evaluate the interaction between the response variable and fluorescence and the rest of the variables measured (see Table 4). Evaluation of the response revealed the same contingency table for all variables. In other words, there was no interaction or confounding factors between the response variable and the rest of the variables. The possibility of a complete response from nBCC to sBCC was reduced by 0.034 times (96.6%) and for the nonspecified type versus sBCC it was reduced by 0.087 times (91.3%). This means only the histological type influenced response. Conversely, on evaluation of the fluorescence result, three different contingency tables were obtained. This means that the size, histological type, and location are confounding factors on BCC fluorescence.

## 4. Discussion

The objective of this study was to describe our center's experience after six years of treating BCC with PDT. We tried to find and describe parameters that would be predictive of response in order to improve selection of BCCs to be treated with PDT and to optimize the technique.

This is a descriptive, retrospective, observations study with less statistical power than clinical trials. However, it reproduces routine clinical practice. The primary limitations of our study were those derived from its retrospective design, compiling patients treated by three different dermatologists and biopsies of the BCCs. Despite its histological diagnosis being simple, they were evaluated by different pathologists. Patients who had more than two BCCs and those who did not complete the standard two-session protocol were excluded. This was done to avoid selecting patients who possibly had undiagnosed Gorlin syndrome, to practically evaluate one lesion per patient and to be able to compare our results with other studies. Nevertheless, there is the possibility of selection bias given that many patients were excluded and BCCs that had a very good response in one session and those that required several sessions but also achieved a complete response were also excluded. The variables for histological type, size, and location were chosen because they are variables that the dermatologist can manage in routine practice. The reason for dividing the location into head (face and neck) and trunk, and the sizes into three groups, was to obtain sufficient statistical power. The precise locations of the BCCs on the face were specified because our group has observed a worse response in some locations. However, we did not have sufficient power to perform an inferential statistical analysis.

The patients treated were older and the majority had moderately sized BCCs on the truck. It is notable that, despite the long follow-up period (34.4 months), the majority of recurrences in our sample occurred in the first six months after treatment, practically making them incomplete responses that end up requiring surgical removal. This may suggest that patients with a complete clinical response do not require a longer follow-up period. However, the guidelines recommend follow-up for one year after treatment [12] and other studies have found more delayed recurrences [4, 7, 13]. One recent retrospective study on 157 BCCs revealed the majority of recurrences in the two years after treatment in 19% of BCCs (26% had incomplete responses or recurrences in our series) [11]. This study also revealed independently that the nodular histological type had the highest rates of recurrences [11].

In our sample, sBCC responded better to PDT overall than nBCC, 95% versus 49%, and this difference was statistically significant (*P* < 0.001). This fact was already noted in another large retrospective study [9] where a complete response rate of 82% was found for sBCC and 33% for nBCC (*P* < 0.000). Together these findings suggest that nBCC is not a good indication for PDT and, as recommended in their treatment guidelines, and surgery continues to be the gold standard for treatment [8]. This finding also calls into question the use of curettage prior to submitting the nBCC to occlusion with the photosensitizer. In our patient group and according to the published recommendations [1], nBCCs are subjected to superficial scraping prior to treatment. Perhaps if this scraping was more intense, we would achieve a better response, though perhaps this procedure would not be PDT but rather curettage plus PDT.

When the lesions are analyzed by location, BCC on the trunk responds better than on the head (86% versus 66%; *P* < 0.001). No association was found between size and the response (*P* < 0.063), but BCCs with exact fluorescence had better complete responses (*P* < 0.029). However, we have a sample in which the sBCCs, which are those that responded best to treatment, were statistically significantly located more in the trunk, their fluorescence is more exact and they were of intermediate size. In other words, it is possible that what was being measured was the same and therefore demonstrated a covariate analysis that revealed that, out of all the variables measured, the only variable that influences response is the histological type. In a 2012 study, Fantini et al. [9] found very similar findings measuring the same variables on 194 BCCs. Nevertheless, when a regression analysis is performed, it is noted that the histological type and the location are independent predictors of response. The study also was of a sample which significantly (*P* < 0.000) consisted of sBCCs located on the trunk.

Regarding BCCs on the face, they are more frequently nodular and small (*P* < 0.05), which accounts for the inferior response to treatment. Regarding the locations, it is noted that some areas have a tendency to have a good or intermediate response such as the scalp, cheeks, temple, forehead, lip, and chin. There are also areas that tend to have a poorer response such as the ears, tip of the nose, and nostril. Clearly one cannot draw conclusions from such a small sample size; however, it is notable that these areas coincide with locations defined as high-risk BCC [8]. High-risk BC is defined as those located in the H zone of the face (eyes, nose, lips, and ears), which coincides with those in our patient group who had a poorer response to PDT.

Fluorescence of the lesions prior to illumination has been a parameter described in many PDT studies as a possible predictive factor for response to treatment. Exact fluorescence in our BCC group is more frequent in lesions that subsequently achieved a complete response (*P* < 0.05). Exact fluorescence is more common in the trunk, sBCCs, and sizes 2 and 3 (*P* < 0.05). These findings appear logical if we think that in the face, where there is more endogenous fluorescence from porphyrins, excessive fluorescence is more frequent and there are more nBCCs which have poorer fluorescence. However, when a covariate analysis of all these variables is performed, it is noted that fluorescence acts as a confounding factor. That is to say, there is no association between fluorescence, histological type, and location and, therefore, there is no association between fluorescence and response. Our study is the only study in the literature that has evaluated the interaction between these factors. There is a previous study that also revealed that there is no association between BCC fluorescence and its response to treatment [10].

## 5. Conclusions

In the treatment of BCC with PDT, the most significant factor for predicting response is the histological type. Superficial BCC responds significantly better than other histological types. BCC fluorescence is influenced by the histological type, the size of the BCC, and the location and cannot predict treatment response. Larger studies are needed in order to evaluate the interaction between all the variables studies and the BCC's response to PDT.

## Figures and Tables

**Table 1:** Clinical caracteristics of the 191 lesions and their *P* values in the trunk-head distribution.

Location	Body 73 (38%) Head 118 (62%)	Body (*n* = 73)	Head (*n* = 118)	*P* = 0.043
Histological type	Superficial 87 (46%)	50 (57%)	37 (43%)	*P* < 0.001
	Nodular 49 (26%)	7 (14%)	42 (86%)	
	Not specified 31 (16%)	10 (32%)	21 (68%)	
	Infiltrating 22 (11%)	6 (27%)	16 (73%)	
	Sclerosing 2 (1%)	0 (0%)	2 (100%)	

Size	Less than or equal to 1 cm: 60 (31%)	5 (8%)	55 (92%)	*P* < 0.001
	Between 1 and 2 cm: 99 (52%)	45 (45%)	54 (55%)	
	Greater than or equal to 2 cm: 32 (17%)	23 (72%)	9 (28%)	

Fluorescence	Exact 99 (52%)	65 (65%)	34 (34%)	*P* < 0.000
	Excessive 79 (41%)	4 (5%)	75 (95%)	
	Defective 13 (7%)	4 (31%)	9 (69%)	

**Table 2:** Response according to the measured variables: location, histological type, size, and fluorescence.

Variable	Response		*P*
	Complete (*n* = 141)	Incomplete (*n* = 50)	
Location			
Trunk (*n* = 73)	63 (86%)	10 (14%)	*P* < 0.001
Head (*n* = 118)	78 (66%)	40 (34%)	
Histological type			
Superficial (*n* = 87)	83/87 (95%)	4/87 (5%)	*P* < 0.001
Not specified (*n* = 31)	22/31 (71%)	9 (19%)	
Nodular (*n* = 49)	24/49 (49%)	24/49 (51%)	
Infiltrating (*n* = 22)	11/22 (50%)	11/22 (50%)	
Morpheaform (*n* = 2)	0/2 (0%)	2/2 (100%)	
Size			
Size 1 (*n* = 60)	41 (68%)	19 (32%)	*P* < 0.063
Size 2 (*n* = 99)	72 (73%)	27 (27%)	
Size 3 (*n* = 32)	28 (87%)	4 (13%)	
Fluorescence			
Exact (*n* = 99)	87 (88%)	12 (12%)	*P* < 0.029
Inexact (*n* = 82)	54 (63%)	38 (47%)	

**Table 3:** Fluorescence pattern based on the variables studied.

Variable	Fluorescence			*P*
	Exact (*n* = 99)	Excessive (*n* = 79)	Defective (*n* = 13)	
Location				
Head (*n* = 118)	34 (29%)	75 (64%)	9 (7%)	*P* < 0.000
Trunk (*n* = 73)	65 (90%)	4 (5%)	4 (5%)	
Histological type				
Superficial (*n* = 87)	63 (72%)	21 (24%)	3 (4%)	*P* < 0.000
Not specified (*n* = 31)	10 (32%)	18 (58%)	3 (10%)	
Nodular (*n* = 49)	19 (39%)	25 (51%)	5 (10%)	
Infiltrating (*n* = 22)	6 (27%)	16 (73%)	0 (0%)	
Size				
Size 1 (*n* = 60)	11 (18%)	45 (75%)	4 (7%)	*P* < 0.000
Size 2 (*n* = 99)	62 (63%)	31 (31%)	6 (93%)	
Size 3 (*n* = 32)	26 (82%)	3 (9%)	3 (9%)	

**Table:** (a) Response (type, size, and location)

Response	OR (95% CI)	*P*
Type		
Superficial	REF	REF
Nodular	0.034 (0.010; 0.123)	<0.001
NS	0.087 (0.022; 0.350)	0.001

**Table:** (b) Fluorescence (type, size, and location)

Fluorescence	OR (95% CI)	*P*
Type location		
Type		
Superficial	REF	REF
Nodular	0.551 (0.233; 1.305)	0.176
NS	0.191 (0.063; 0.576)	0.003
Size		
Size 3	REF	REF
Size 1	5.963 (2.394; 14.849)	<0.001
Size 2	19.521 (108.963);	0.001

Size type		
Size		
Size 3	REF	REF
Size 1	10.536 (4.524; 24.534)	<0.001
Size 2	60.000 (11.990; 300.251)	<0.001
